# Metastatic extension of Ewing’s sarcoma to the right heart chambers: a rare case report

**DOI:** 10.1186/s43044-025-00619-1

**Published:** 2025-02-25

**Authors:** Yassine Ettagmouti, Salah-Eddine Hayar, Ilyas Atlas, Ghita Bennani, Meryem Haboub, Rachida Habbal

**Affiliations:** 1 Cardiology Division, Ibn Rochd University Hospital, Casablanca, Morocco; 2 Radiology Division, 20 AOUT 1953 Hospital, Casablanca, Morocco

## Abstract

**Background:**

Ewing’s sarcoma (ES) is a common malignant bone tumor in adolescents and young adults. Its pelvic location is associated with a worse prognosis. Our case represents one of the rare instances in the literature involving an adult patient in whom the disease progressed fatally due to cardiac extension.

**Case presentation:**

We report the case of a 31-year-old female patient who initially presented with swelling in her right lower extremity, which was found to be caused by deep venous thrombosis (DVT) extending from the iliac vein to the inferior vena cava. A thoracic-abdominal CT scan, performed as part of the etiological workup, revealed a tumor in the right hip bone with a malignant appearance, exhibiting both endo and exopelvic extension, and extending to the inferior vena cava (IVC) and right heart chambers. An echo-guided biopsy of the tumor mass confirmed Ewing's sarcoma. The patient's condition rapidly deteriorated, leading to death due to the inoperability of the extensive tumor.

**Conclusions:**

Ewing’s sarcoma can affect adults, presenting with late-onset or rapidly metastatic forms. In its extensive form, ES requires multimodal imaging to assess operability and is associated with a poor prognosis. This case report represents one of the rare instances in the literature of Ewing’s sarcoma metastasizing to the heart.

## Background

Ewing’s sarcoma (ES) is the second most common malignant bone tumor after osteosarcoma in adolescents and young adults, with a particularly poor prognosis when located in the pelvis. The majority of cases occur in individuals aged 5–25 years [1].

Our case illustrates a late diagnosis of ES in a 30-year-old patient, characterized by a tumor thrombus extending from the inferior vena cava to the right atrium and into the right ventricle.

## Case presentation

A 30-year-old female patient, with no significant medical history and using estroprogestative contraception, presented to the emergency department with right leg swelling that had been evolving for 3 weeks.

Physical examination revealed a tender, swollen, erythematous right limb extending from the ankle to the hip, raising suspicion of deep venous thrombosis. We performed venous ultrasounds, which confirmed an extensive deep venous thrombosis extending from the right iliac vein to the inferior vena cava.

An echocardiogram was conducted, revealing a right atrial mass suggestive of a right atrial thrombus, consistent with the context of proximal deep venous thrombosis. Anticoagulation therapy was initiated using enoxaparin at a dose of 100 IU/kg twice daily.

To assess for concomitant pulmonary embolism and determine the etiology, we ordered a thoracic and abdominal CT angiogram. While pulmonary embolism was excluded, the scan revealed a tumoral process in the right coxal bone with malignant characteristics, including endo- and exopelvic extension, suggestive of Ewing's sarcoma.

**Fig. 1:**
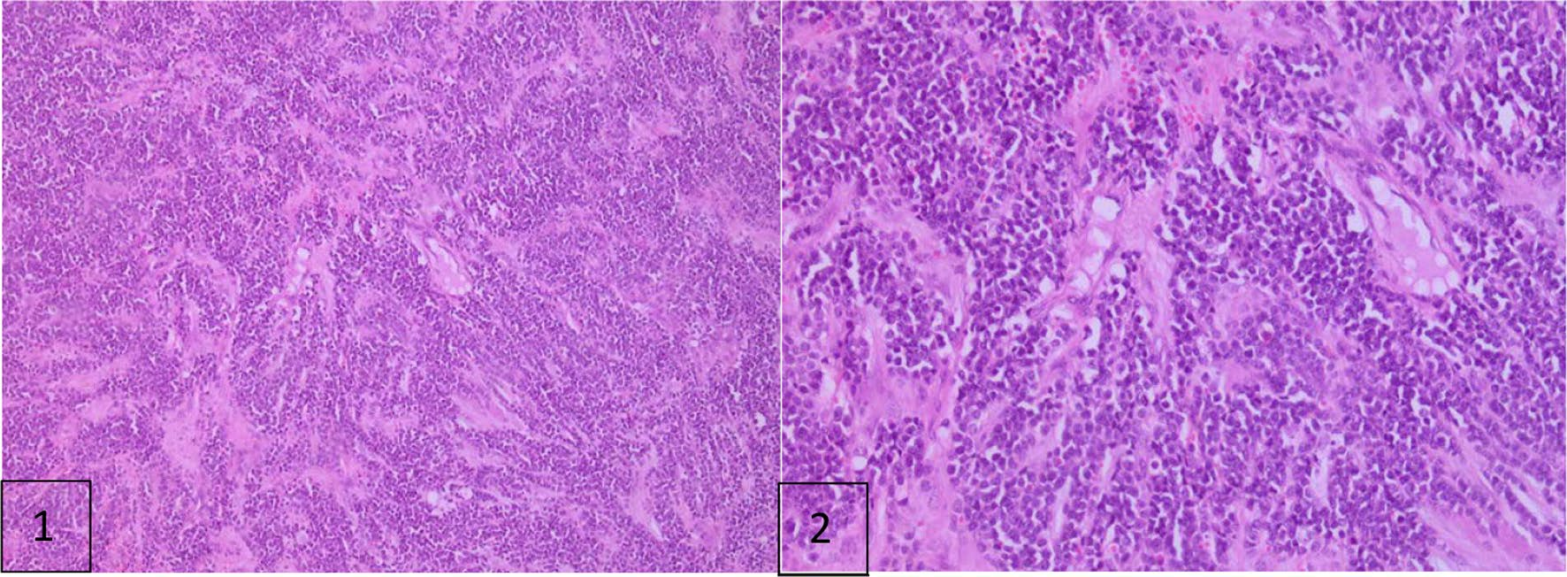
Optical microscopic magnificiance H & e: **1** × 10 tumoral proliferation organized in layers; **2** the cells are rounds, medium sized, with poorly defined cytomplasm

A biopsy confirmed Ewing’s sarcoma, characterized by medium-sized rounded tumoral cells proliferating in sheets (Fig. 1). Consequently, the patient was proposed for neoadjuvant chemotherapy.

One month later, the patient returned to our department with worsening symptoms, including stage III NYHA dyspnea of rapid onset, palpitations, and bilateral lower limb swelling. In response, another venous Doppler ultrasound was performed, revealing extensive DVT from the popliteal vein to the left common femoral vein.

**Fig. 2:**
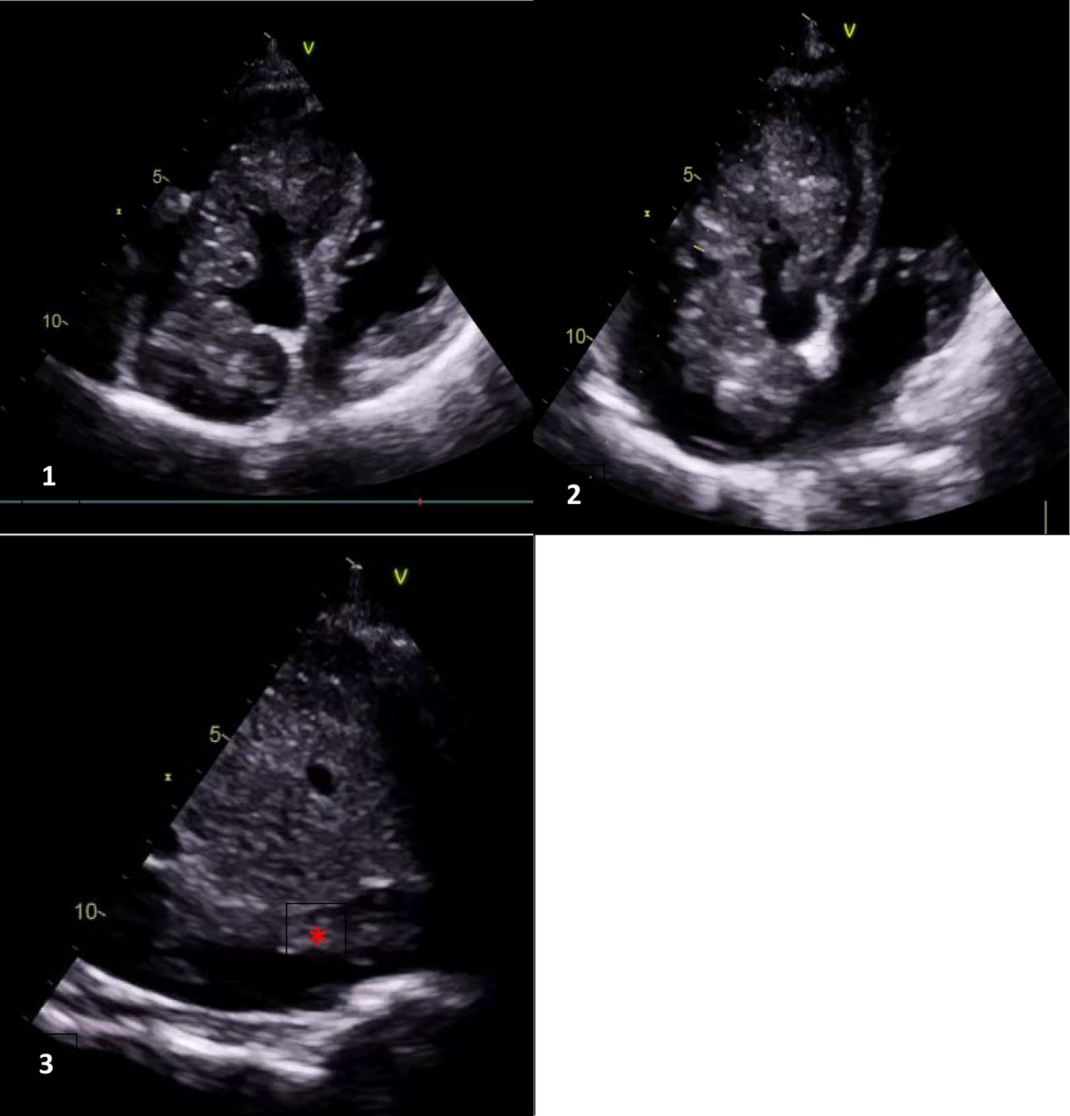
Transthoracic echocardiography: **1**–**2** 4 chambers view: polylobed echogenic mass, mildly calcified hanging between the right atrium and the right ventricle through the tricuspid valve, measuring 70 mm along its length, obstructing intermittently the tricuspid flow. **3** Sub costal view, Red Asterix: tumoral extension through the inferior vena casa

Upon repeated echocardiography, we observed a dilated right ventricle with preserved longitudinal systolic function, along with a large, polylobulated echogenic mass that was mildly calcified and had a tumoral appearance. This mass was hanging between the right atrium and the right ventricle, measuring 70 mm in length, and was intermittently obstructing tricuspid flow with a mean gradient of 8 mmHg. It was continuous with a similar echogenic structure in the inferior vena cava, raising suspicion of continuous transvenous metastasis of the Ewing sarcoma from the coxal bone to the right heart chambers (Fig. 2).

**Fig. 3:**
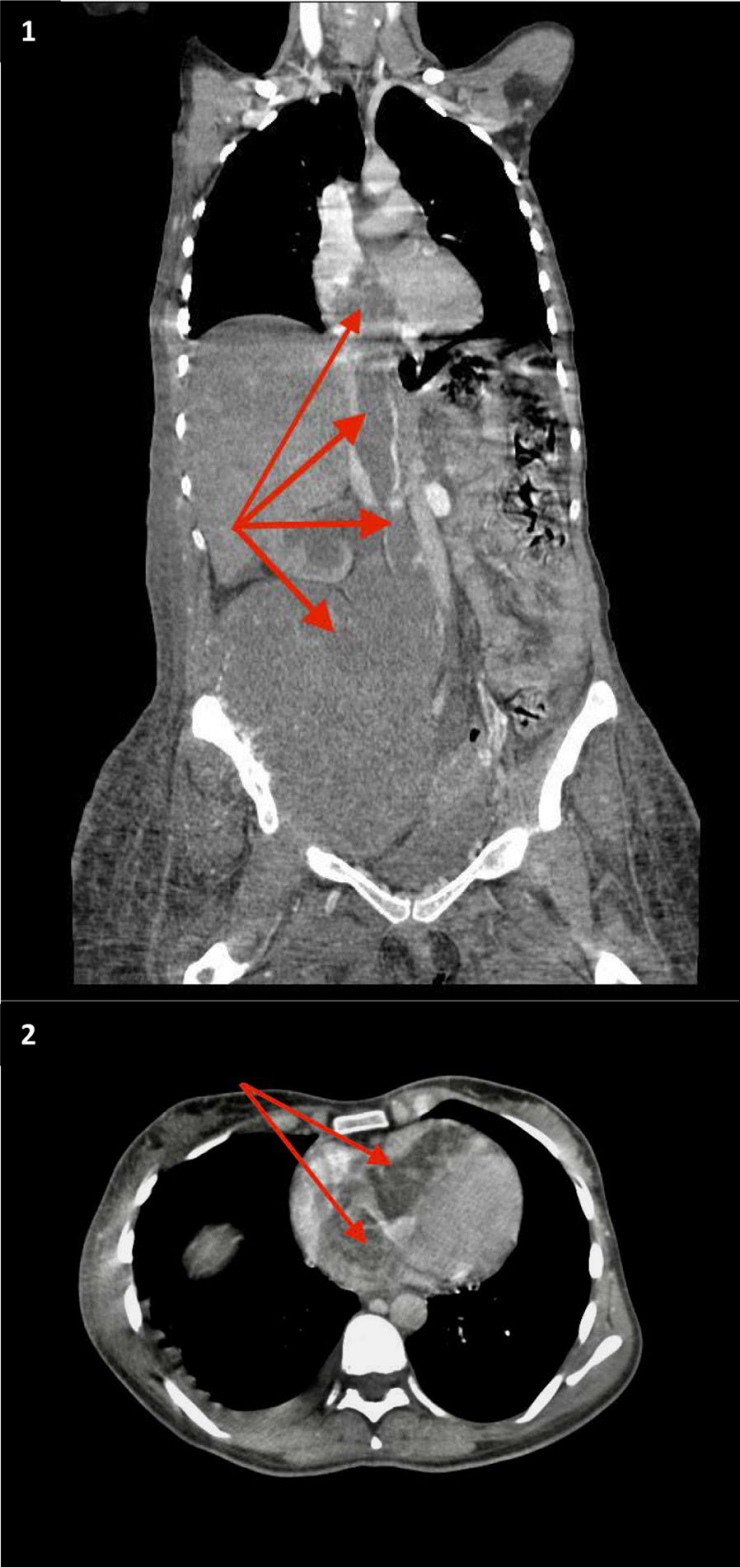
**1** Thoraco abdominal angio graphy computed tomography: coronal view: large lesional process of the right coxal bone with endo and exopelvic development associated with bone lysis and invading the IVC which is partially thrombosed with the mass of the same characteristics in favor of a tumoral extention to the right heart chambers. **2** Thoracic angiography computed tomography, transversal view: hypodense mass obliterating right heart chambers. Red arrows: Tumor extension into the IVC and right cavities

A repeated thoraco-abdominal CT scan confirmed the continuity between the primary tumor and the right heart through the vena cava. The scan revealed a large lesional process in the right coxal bone, measuring 19 × 16 cm with a height of 18 cm, associated with bone lysis and invading the inferior vena cava, which was partially thrombosed. A mass of similar characteristics to the right coxal mass was observed, extending into the right heart chambers without visible material in the lumen of the pulmonary artery (Fig. 3).

The case was discussed in a multidisciplinary meeting, including oncologists, cardiologists, and cardiac surgeons, who concluded that the patient was inoperable and recommended palliative care. The patient passed away three weeks later.

## Discussion

Metastases can reach the heart through various routes, including direct extension, hematogenous spread, lymphatic spread, or intra-cavity diffusion via the vena cava or pulmonary veins. The right side of the heart is more frequently affected than the left side [4]. Bone sarcomas rarely lead to cardiac metastases, typically disseminating to the heart through the hematogenous route [5].

Genetic, molecular, and immunological mechanisms [11, 12] influence the metastasis and dissemination of Ewing's sarcoma. Among these mechanisms, we can cite:

- Mutations that lead to the degradation of components of the extracellular matrix.
- Activation of signaling pathways and angiogenesis.
- Immunosuppression and escape from positive regulation by T lymphocytes and macrophages.

These mechanisms have therapeutic implications for the prognosis of Ewing's sarcoma and have paved the way for targeted treatments in the future.

The incidence of secondary cardiac tumors is reported to be between 0.7 and 3.5% in autopsy studies of the general population [6]. The prognosis for Ewing’s sarcoma in the pelvis, regardless of tumor metastasis, is poor. Treatments such as chemotherapy, radiotherapy, and surgery for pelvic Ewing’s sarcoma have resulted in a 5-year survival rate of only 40% [9].

In a cohort of 451 patients with primary pelvic bone tumors, the prevalence of tumor-associated venous thrombosis was found to be 9.8%. Factors associated with the development of tumor thrombosis included a lactate dehydrogenase (LDH) level of ≥ 230.5 U/L and invasion of the L5-S1 intervertebral foramen [3].

High serum LDH levels are considered a poor prognostic factor in Ewing’s sarcoma and are included in the International Prognostic Index [15]. Elevated LDH has been associated with advanced disease, increased tumor burden, and poorer overall survival.

We presented a case of Ewing’s sarcoma originating from the right coxal bone, which led to the formation of a tumor thrombus in the inferior vena cava that extended to the right atrium and subsequently to the right ventricle.

The primary purpose of imaging is to differentiate between a thrombus and a tumor while awaiting histological confirmation, if feasible. Echocardiography serves as the basic examination for diagnosing these cardiac tumors.

The use of additional imaging techniques, such as computed tomography (CT) or MRI, is common. MRI is particularly relevant for studying a cardiac mass due to its high resolution, allowing for detailed assessment of its composition and dynamic and late enhancement characteristics. While CT provides less optimal tissue contrast, it offers better definition of the anatomical relationships with adjacent structures [8].

Thus, Differentiation between a tumoral thrombus and a fibrinocruoric thrombus can be challenging [16]; however, imaging may be helpful in showing the following:

- *Tumoral thrombus* often located in blood vessels near the primary tumor or metastases. On ultrasound, the contours are irregular, with visible intratumoral neovascularization on Doppler imaging. On cross-sectional imaging, the mass appears heterogeneous, with areas of necrosis or enhancement, indicating tumor blood supply.
- *Fibrinocruoric thrombus* often located in the deep veins of the lower limbs or in areas of disturbed blood flow. On imaging, the thrombus appears homogeneous in density, composed of fibrin and platelets according to its age, and does not enhance after contrast injection, unlike the tumoral thrombus.

Advances in PET-CT and advanced MRI protocols have greatly improved the management of Ewing’s sarcoma [14]: PET-CT provides both anatomical and metabolic information, which is essential for identifying the primary tumor, detecting metastases, and staging the disease. It also allows for assessing the efficacy of chemotherapy and detecting recurrences in difficult-to-evaluate areas, offering a comprehensive view of disease spread. Whole-body MRI can be a valuable alternative to PET-CT, especially for assessing bone marrow involvement and soft tissue metastases in the pediatric population. Dynamic contrast-enhanced MRI assesses tumor vascularity and angiogenesis, which are crucial for evaluating the aggressiveness of the tumor.

In our case, the scannographic appearance of the thrombus was of similar density to that of the primary tumor, indicating that the thrombosis was tumoral rather than fibrinous. In this context, anticoagulation would be ineffective; consequently, chemotherapy coupled with radiotherapy was proposed for the patient. Unfortunately, there was no significant improvement, and the patient experienced a rapid decline, ultimately leading to death.

Previous articles have shown no significant difference in the outcomes of patients with pelvic Ewing’s sarcoma treated with surgery or radiation [10]. A recent international working group consensus, published in 2022, implemented dose-intensified, short-interval aggressive chemotherapy regimens that demonstrated improved event-free survival in patients with localized Ewing sarcoma. Future prospects for molecular therapies, including combination modalities and gene sequencing, are currently under investigation [13].

In this case, if curative surgery was to be performed, both amputation of the right hip and evacuation of the tumor from the inferior vena cava and right heart chambers would be necessary. This would be a very complicated and risky procedure for the patient.

A case reported in the literature involving Ewing's sarcoma with a tumor thrombus in the IVC was managed successfully with radiotherapy and chemotherapy without resorting to surgery [2]. This approach may be a viable alternative before the thrombosis extends to the heart chambers. If there is metastasis, intensive chemotherapy or molecular therapies if available on a case-by-case basis.

The rapid death of the patient may be attributed to adiastole with filling failure of the right ventricle secondary to the large size of the tumor and blockage of the tricuspid valve movement, or to tumor cell embolization leading to massive pulmonary embolism [7].

## Conclusions

Key takeaways from this case include:

1. Ewing’s sarcoma can also affect adult patients, presenting with late onset or rapidly metastatic forms.
2. Localized pain or swelling in the bones or soft tissues, generalized fever, deterioration of the general condition, and dyspnea are key signs that may indicate tumor progression.
3. Advances in multimodal imaging (CT, MRI, PET-CT) have significantly improved the early detection and monitoring of metastasis in Ewing’s sarcoma.
4. LDH is a key indicator of tumor activity and treatment response.
5. The combination of chemotherapy and radiotherapy may serve as an effective therapeutic solution, potentially avoiding the need for surgery.
6. The advent of molecular therapies offers new perspectives for the management of Ewing’s sarcoma.

This case report is one of the rare instances in the literature documenting tumor metastasis to the heart in Ewing’s sarcoma.

## Data Availability

No datasets were generated or analyzed during the current study.

## Abbreviations

ES: Ewing’s sarcoma
DVT: Deep venous thrombosis
IVC: Inferior vena cava
NYHA: New York heart association
LDH: Lactate dehydrogenase
CT: Computed tomography
MRI: Magnetic resonance imaging
